# Airway management in acutely poisoned patients with Glasgow Coma Scale less than 9: a retrospective cohort study

**DOI:** 10.1186/s12873-026-01714-5

**Published:** 2026-08-06

**Authors:** Sabrina Schmoll, Eva Mehrl, Stefanie Geith, Florian Eyer, Christian Rabe, Tobias Zellner

**Affiliations:** Department of Clinical Toxicology and Poison Center Munich, Department of Internal Medicine II, TUM School of Medicine and Health, Technical University of Munich, TUM University Hospital Munich, Ismaninger Str. 22, 81675 Munich, Germany

**Keywords:** Acute poisoning, Glasgow Coma Scale, Airway management, Intubation, Toxicology

## Abstract

**Background:**

A Glasgow Coma Scale (GCS) ≤ 8 is frequently used to guide prehospital intubation, but its generalizability to acutely poisoned patients is uncertain. We compared outcomes of poisoned patients with GCS ≤ 8 managed with or without prehospital intubation.

**Methods:**

We retrospectively included poisoned patients with a GCS ≤ 8 found as helpless persons in public settings and admitted to a tertiary toxicology center. Patients were grouped by prehospital intubation status. Clinical characteristics, management, and outcomes were analyzed using nonparametric and categorical tests, the analyses were exploratory due to the limited sample size.

**Results:**

Among 84 patients, 10 (11.9%) were intubated prehospitally, and 74 (88.1%) were not. Median GCS was lower in intubated patients (3 vs. 7). Aspiration pneumonitis in non-intubated patients was rare (6.8%) and did not differ significantly between groups. Vasopressor therapy within 24 h was required in one of 74 (1.4%) non-intubated patients and two of 10 (20.0%) intubated patients. Antidotes were frequently administered, especially in non-intubated patients, predominantly in the prehospital setting. Two non-intubated patients (2.7%) required delayed mechanical ventilation. No deaths occurred.

**Conclusions:**

Non-intubated patients showed favorable outcomes with low complication rates and infrequent delayed intubations, while antidotes were frequently administered in both the prehospital and in-hospital settings. These results suggest that GCS alone may be insufficient to guide airway management in poisoning, supporting an individualized, clinically guided approach.

**Supplementary Information:**

The online version contains supplementary material available at 10.1186/s12873-026-01714-5.

## Background

While a Glasgow Coma Scale (GCS) score ≤ 8 is widely used as a threshold for guiding intubation in traumatic brain injury, its application toward the care of poisoned patients remains uncertain [1–4].

In patients with acute poisoning, reduced consciousness may result from rapidly reversible pharmacological effects rather than structural neurological injury. Consequently, the decision to perform endotracheal intubation is often more complex than in patients with traumatic brain injury.

The principal rationale for intubation in poisoned patients is the prevention of airway compromise and aspiration pneumonitis. Clinicians must balance this potential benefit against the risks associated with invasive airway management, including procedural complications, hemodynamic instability, prolonged mechanical ventilation, intensive care admission, and increased healthcare resource utilization. Reduced consciousness alone may not reliably predict aspiration or respiratory failure in toxicological patients, particularly when airway reflexes and spontaneous ventilation are preserved.

Recent evidence has challenged the routine use of GCS-based intubation thresholds in poisoning. In a multicenter randomized clinical trial, Freund et al. demonstrated that a restrictive airway-management strategy, reserving intubation only for patients who developed complications such as hypoxemia, seizures, vomiting, or respiratory insufficiency, was associated with favorable outcomes and reduced resource utilization compared with routine airway management [5]. Similarly, observational studies have questioned whether a low GCS score alone should mandate intubation in poisoned patients [3–8].

There is still uncertainty about which patients with severe poisoning and depressed consciousness can safely be managed without immediate airway intervention. In particular, real-world data describing patients managed according to physician judgment in routine prehospital practice are limited. Therefore, we aimed to compare the characteristics and outcomes of a clearly defined subgroup of acutely poisoned patients with GCS ≤ 8 who were managed with versus without prehospital intubation. This subgroup is comparable to the cohort described by Freund et al. [5] and could serve as a cohort for the prospective study of intubation strategies in upcoming trials. Given the retrospective, observational design, our objective was not to determine the efficacy of intubation but rather to describe current practice and to generate hypotheses regarding airway management decisions in this population.

### Methods and participants

This retrospective comparative cohort study was conducted at a tertiary academic toxicology center serving approximately 1.5 million inhabitants. The center includes a regional poison information service, an intermediate care unit, and a specialized toxicological intensive care unit. We included all acutely poisoned adult patients (≥ 18 years) who were found as helpless persons in public settings and subsequently admitted to our institution from January 2016 to December 2017, before the publication of Freund et al.’s study.

Acute poisoning was diagnosed based on the clinical assessment by the treating emergency physician and toxicologist, taking into account the patient’s history when available, witness reports, characteristic clinical findings, toxicological analyses, and/or the response to antidote administration. Patients with single-substance intoxications, mixed poisonings, and poisonings involving unidentified substances were eligible for inclusion.

Patients were included if they had a Glasgow Coma Scale (GCS) score ≤ 8 at first medical assessment, either in the prehospital setting or upon arrival at the emergency department. Patients were grouped according to whether prehospital endotracheal intubation had been performed.

Prehospital care was provided within a physician-staffed emergency medical service system. Decisions regarding airway management were made by the treating emergency physician based on clinical judgment and local practice, without a standardized airway management protocol.

Antidote administration was guided by suspected toxidrome and occurred before any decision regarding airway intervention whenever feasible. Naloxone and flumazenil were administered according to standard emergency medical services protocols, based on the treating physician’s clinical judgment.

The term “helpless person” refers to a predefined operational category used within the regional emergency medical services documentation system. It describes individuals found in public spaces who were unable to care for themselves because of impaired consciousness or functional incapacity and therefore required emergency medical evaluation.

### Exclusion criteria

Patients were excluded if they were younger than 18 years of age, found in private residences, or had an alternative primary diagnosis explaining reduced consciousness (e.g., traumatic brain injury).

Patients with cardiac arrest before hospital admission were also excluded. Patients with mixed poisonings or unidentified substances were not excluded, provided that acute poisoning was considered the primary diagnosis by the treating physicians.

### Data collection

Data were obtained from electronic hospital records and standardized prehospital emergency medical service documentation. Variables collected included demographic characteristics (age, sex), clinical parameters (Glasgow Coma Scale at first assessment), type of substance exposure, prehospital interventions (including airway management and antidote administration), and in-hospital outcomes. GCS scores were assessed by trained medical personnel in the prehospital or emergency department setting. No formal sample size calculation was performed due to the retrospective and exploratory nature of the study. All eligible patients within the study period were included.

### Outcomes

The primary outcome was aspiration pneumonitis. Aspiration pneumonitis was defined as the presence of new pulmonary infiltrates on chest imaging and/or initiation of antibiotic therapy for clinically suspected aspiration during the hospital stay. Chest radiographs were independently evaluated by three experienced intensive care physicians. The diagnosis of aspiration pneumonitis was also established by majority consensus. Because of the retrospective design, differentiation between chemical aspiration pneumonitis and bacterial aspiration pneumonia was not possible.

Outcome assessment was based on a review of clinical records, radiological reports, and treatment documentation from admission until hospital discharge.

Secondary outcomes included in-hospital mortality, the need for procedures that could only be performed in the ICU (mechanical ventilation, vasopressors), and length of hospital stay.

### Data analysis

All analyses were performed using standard statistical software (SPSS version 31.0.0.0, IBM Corp., Armonk, NY, USA).

Continuous variables are reported as median (range) and were compared using the Mann–Whitney U test. Categorical variables are presented as counts and proportions and were compared using the chi-square test or Fisher’s exact test, as appropriate. For binary outcomes, exact (Clopper–Pearson) 95% confidence intervals were calculated for the observed proportions. Given the exploratory nature and limited sample size of the study, all analyses were considered descriptive and hypothesis-generating. Absolute risks and corresponding 95% confidence intervals were prioritized for the interpretation of binary outcomes. Where appropriate, exploratory effect estimates (odds ratios) were also reported. No adjustment for multiple comparisons was performed. A two-sided p value < 0.05 was considered statistically significant.

Missing data were minimal, and no imputation was performed.

This study is reported in accordance with the STROBE guidelines.

An artificial intelligence-based tool (Grammarly, Superhuman Platform Inc, San Francisco, CA, USA) was used to check spelling, style, grammar, and punctuation.

## Results

**Fig. 1:**
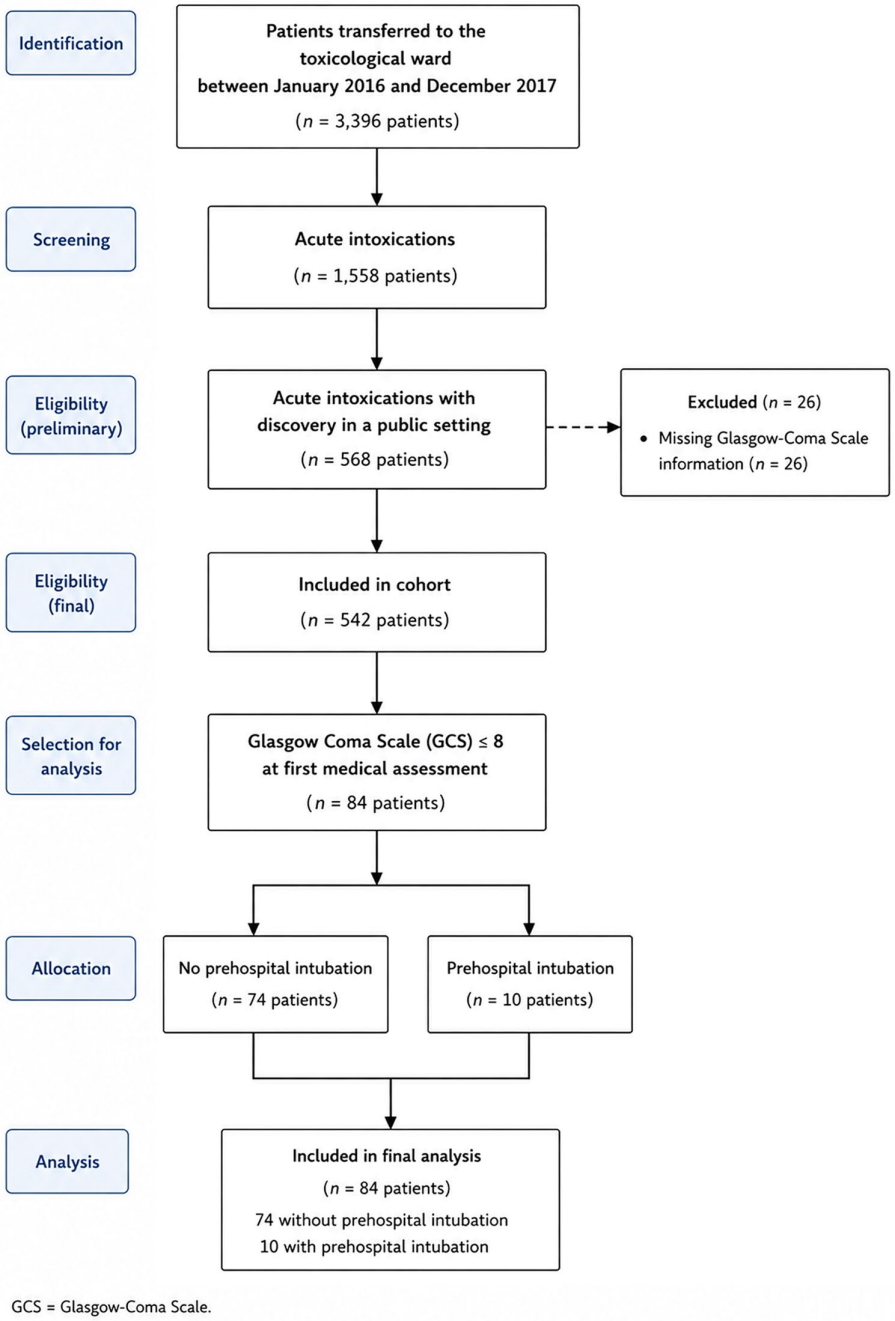
Flow diagram of patient selection. Patients admitted to the toxicology ward between January 2016 and December 2017 were screened for eligibility. Patients with acute poisoning who were found in public settings were assessed for inclusion. After exclusion of patients with missing Glasgow Coma Scale (GCS) data, 84 patients with acute poisoning and a GCS score ≤ 8 were included in the final analysis

Among 542 poisoned patients found as helpless persons in public spaces, 84 (15.5%) had a GCS ≤ 8. Of these, 74 (88.1%) were not intubated, and 10 (11.9%) were intubated and included in the analysis (Table 1; Fig. 1. Study flow diagram).

Baseline physiological parameters were broadly similar between groups. No statistically significant differences were observed in oxygen saturation, systolic blood pressure, diastolic blood pressure, heart rate, or respiratory rate at first assessment (all *p* > 0.05). Complete blood gas and laboratory findings are summarized in Supplementary Table S1. In contrast, the intubated group had significantly lower GCS scores, indicating greater neurological impairment at presentation (Table 2).

**Table 1:** Baseline characteristics and outcomes of patients with GCS ≤ 8 according to airway-management group

Variable	No prehospital intubation (*n* = 74)	Prehospital intubation (*n* = 10)	Significance (*p*-value)
Male sex, n (%)	47 (63.5%)	8 (80.0%)	0.48¹
Age, years	31 (18–58)	35 (21–58)	0.64²
Hospital stay, days	1 (0–11)	2.5 (0–14)	0.21²
GCS score	7 (3–8)	3 (3–8)	< 0.001²
Aspiration pneumonitis, n (%)	5 (6.8%; 95% CI 2.2–15.1%)^3^	0 (0.0%; 95% CI 0.0-30.8%)^3^	1.00¹
Antidote therapy, n (%)	19 (25.7%; 95% CI 16.2–37.2%)^3^	1 (10.0%; 95% CI 0.3–44.5%)^3^	0.42¹
Mortality, n (%)	0 (0.0%)	0 (0.0%)	—

^1^Fisher’s exact test (used due to small expected cell counts).

^2^ Mann–Whitney U test (Continuous variables are presented as median (range) Categorical variables as n (%)).

^3^Exact (Clopper–Pearson) 95% confidence intervals for proportions

**Table 2:** Vital signs according to airway-management group

Variable	No prehospital intubation (*n* = 74)	Prehospital intubation (*n* = 10)	*p*-value
Oxygen saturation (%)	97 (78–100)	97 (94–100)	0.771
Systolic blood pressure (mmHg)	114 (80–167)	122 (91–160)	0.525
Diastolic blood pressure (mmHg)	68 (50–114)	76 (48–91)	0.237
Heart rate (beats/min)	90 (60–141)	80 (49–105)	0.216
Respiratory rate (/min)	12 (5–24)	14 (8–18)	0.366

Values are presented as median (range). Comparisons were performed using the Mann-Whitney U test

Of the 84 patients, 74 were not intubated prehospitally despite a GCS of 8 or lower. Antidotes were administered to 19 of 74 patients (25.7%), predominantly in the prehospital setting (73.6%). One was treated with flumazenil, 14 with naloxone, and 4 were treated with both flumazenil and naloxone. One of 10 prehospitally intubated patients (10.0%) received naloxone and flumazenil before intubation. In one case, a laryngeal tube was inserted prehospital because of profound central nervous system depression. Following naloxone administration, the patient’s level of consciousness rapidly improved, spontaneous ventilation recovered, and the patient arrived at the hospital awake without the need for endotracheal intubation. The odds ratio for antidote administration was 3.33 (95% CI 0.40–27.8; *p* = 0.42), indicating no statistically significant difference between groups. Two patients (2.7%) required delayed mechanical ventilation within 24 h. Detailed clinical characteristics of the patients requiring delayed intubation are provided in Supplementary Table S2.

No deaths occurred. Vasopressor therapy within the first 24 h was required in one of 74 (1.4%) non-intubated patients and two of 10 (20.0%) prehospitally intubated patients (OR 18.3, 95% CI 1.5–224.3; Fisher’s exact test, *p* = 0.036; Supplementary Table S3). Given the very small number of events, this finding should be interpreted with caution and considered exploratory only.

Aspiration pneumonitis was observed in five of 74 non-prehospitally intubated patients (6.8%; 95% CI 2.2–15.1%) including both of the delayed intubations within 24 h. One of the five patients was newly diagnosed as a case of aspiration pneumonitis after independent review of the chest radiograph by three experienced intensive care physicians, with the final diagnosis established by majority consensus. None of the 10 preclinically intubated patients (0%; 95% CI 0.0-30.8%) was diagnosed with aspiration pneumonitis. Given the small number of events and the limited sample size, no reliable conclusions regarding differences between groups can be drawn.

The distribution of poisoning agents was similar between groups (Supplementary Table S4). Mixed intoxications were the most common poisoning category, accounting for 43 of 84 cases (51.2%), followed by ethanol intoxication (38.1%). Approximately half of the intubated patients had mixed intoxications (50.0%), while the remaining cases were attributed to ethanol intoxication.

No statistically significant difference in hospital length of stay was observed between groups (Table 1).

## Discussion

In this retrospective selected cohort of acutely poisoned patients with a Glasgow Coma Scale (GCS) ≤ 8 who were not intubated prehospitally, clinical outcomes were favorable. No deaths occurred, only two patients required delayed mechanical ventilation and vasopressor therapy within the first 24 h was infrequent. The incidence of aspiration pneumonitis was low (6.8%). No significant difference between groups was observed (*p* = 1.00), although a small sample size limited the analysis. These findings indicate that many patients who were selected by treating physicians for non-invasive airway management experienced favorable outcomes. However, because airway-management decisions were based on clinical judgment, the present data cannot determine whether similar outcomes would have occurred under alternative management strategies [4, 6–8]. In line with our data, Freund et al. demonstrated that a similar restrictive strategy—reserving intubation for patients with respiratory compromise, vomiting, or seizures—may reduce complications without compromising safety [5]. Smaller observational studies have similarly questioned the routine application of trauma-derived GCS thresholds to poisoned patients, noting that decreased consciousness in toxicological conditions is often transient and reversible [8, 9]. Aspiration pneumonitis remains the primary concern with an unprotected airway. The observed aspiration rate was comparable to that reported in the restrictive airway-management group described by Freund et al. [5]. However, because of the limited sample size and observational design, our data do not permit conclusions regarding the comparative risks of different airway-management strategies.

A notable feature of our cohort was the frequent administration of antidotes, with over one-quarter of patients receiving therapy, predominantly in the prehospital setting. These findings are consistent with a pragmatic clinical approach that combines careful clinical monitoring with targeted pharmacological reversal, when appropriate, before proceeding to invasive airway management.

In patients with suspected traumatic brain injury, early airway protection remains essential. Beyond individual patient care, a selective airway-management strategy may also have implications for resource utilization, as endotracheal intubation is closely associated with intensive care admission and higher healthcare costs.

This study is limited by its retrospective, observational design, which introduces potential selection bias and unmeasured confounding, particularly as airway management decisions were based on clinical judgment rather than a standardized protocol. The small sample size—especially in the intubated group—and low event rates limit statistical power and the precision of estimates. The absence of a control group precludes causal inference. Additionally, incomplete clinical data at the time of the decision whether to intubate may have influenced management and outcomes. Patients in the intubated group had substantially lower GCS scores, indicating a more severe clinical presentation. Therefore, confounding by indication must be assumed.

The definition of aspiration pneumonitis represents an additional limitation. Because outcome assessment was based on retrospective review of clinical documentation, distinguishing chemical aspiration pneumonitis from aspiration pneumonia was not always possible. Consequently, some degree of outcome misclassification may have occurred. Nevertheless, aspiration-related pulmonary complications were chosen as the primary outcome because aspiration prevention remains the main clinical justification for airway protection and endotracheal intubation in poisoned patients with impaired consciousness.

Similarly, the observed difference in vasopressor requirements should be interpreted with caution because it was based on only three events, resulting in considerable statistical uncertainty despite the nominal statistical significance.

Furthermore, the restriction of the study population to patients classified as helpless persons in public settings may have introduced selection bias and limited the external validity of our findings, as the results may not be generalizable to intoxicated patients encountered in other clinical or prehospital contexts.

Finally, as a single-center study conducted in a specialized toxicology setting with predominantly alcohol- and drug-related intoxications, generalizability to other populations and settings may be limited. Our findings should be considered descriptive and hypothesis-generating and do not permit conclusions regarding the comparative effectiveness or safety of different airway-management strategies.

## Conclusions

In this retrospective cohort, most acutely poisoned patients with GCS ≤ 8 who were not selected for immediate prehospital intubation experienced favorable outcomes, including low rates of aspiration pneumonitis and no mortality. However, due to the observational design, small sample size, and confounding by indication, no conclusions can be drawn regarding the comparative effectiveness or safety of different airway-management strategies. These findings support the need for an individualized approach to airway management rather than relying solely on a GCS-based intubation threshold in acutely poisoned patients. Further prospective studies are needed to better define criteria for airway management in this population and to support evidence-based decision-making.

## Supplementary Information

Below is the link to the electronic supplementary material.

Supplementary Material 1

Supplementary Material 2

## Data Availability

The datasets and materials generated and/or analysed during the current study are not publicly available due to data protection regulations and the sensitive nature of patient health data but are available from the corresponding author on reasonable request.
