# Supplementary material for: Airway management in acutely poisoned patients with Glasgow Coma Scale less than 9: a retrospective cohort study
Source: BMC Emerg Med. 2026 Aug 6;26:215. doi: 10.1186/s12873-026-01714-5 (PMC13445855; doi:10.1186/s12873-026-01714-5)
Supplement: Supplementary file 1 [file 12873_2026_1714_MOESM1_ESM.docx]

Title and Abstract

1. Title and abstract
(a) Indicate the study’s design with a commonly used term
→ The study design (“retrospective cohort study”) is stated in the title.

(b) Provide an informative and balanced summary
→ The abstract summarizes background, methods, results, and conclusions, and reflects the exploratory nature of the analyses.

Introduction

2. Background/rationale
→ The introduction explains the clinical relevance of GCS-based intubation thresholds and their uncertain applicability in poisoned patients.

3. Objectives
→ Clearly stated: to compare outcomes of acutely poisoned patients with GCS ≤8 managed with versus without prehospital intubation.

Methods

4. Study design
→ Retrospective comparative cohort study; stated at the beginning of the Methods section.

5. Setting
→ Tertiary academic toxicology center in Munich, Germany, including description of catchment area and specialized care structures.
→ Prehospital care provided by a physician-staffed emergency medical service system.

6. Participants
(a) Eligibility criteria
→ Inclusion: acutely poisoned patients, GCS ≤8, found as helpless persons in public settings.
→ Exclusion: alternative causes of impaired consciousness (e.g., traumatic brain injury), patients found in private residences.

(b) Methods of selection
→ All consecutive eligible patients during the study period were included; no sampling was performed.
→ Patients identified through hospital records and prehospital emergency medical service documentation.

7. Variables
→ Exposure: prehospital intubation status
→ Primary outcome: aspiration pneumonitis
→ Secondary outcomes: in-hospital mortality, ICU-level interventions (mechanical ventilation, vasopressors), and length of hospital stay

8. Data sources/measurement
→ Data obtained from electronic hospital records and standardized prehospital emergency medical service documentation.
→ GCS scores assessed by trained medical personnel.
→ Definitions (e.g., aspiration pneumonitis) are provided.

9. Bias
→ Potential sources of bias include retrospective design, selection bias, and confounding by indication due to non-standardized airway management decisions.

10. Study size
→ All eligible patients within the study period were included.
→ No formal sample size calculation was performed due to the exploratory nature of the study.

11. Quantitative variables
→ Continuous variables summarized as medians (ranges); categorical variables as counts and percentages.

12. Statistical methods
(a) Statistical tests described (Mann–Whitney U test, chi-square test, Fisher’s exact test)
(b) No multivariable analysis performed due to limited sample size and low number of outcome events
(c) Missing data were minimal and no imputation was performed
(d) Analyses were considered exploratory and hypothesis-generating

Results

13. Participants
(a) Number of patients at each stage described (542 screened → 84 included)
(b) Reasons for exclusion described
(c) A flow diagram illustrating patient selection is provided

14. Descriptive data
→ Baseline characteristics (e.g., age, sex, GCS) are reported in Table 1.

15. Outcome data
→ Outcomes reported for both groups, including aspiration pneumonitis, mortality, and ICU-level interventions.

16. Main results
(a) Group comparisons presented with p-values
(b) No adjusted estimates reported
(c) Results interpreted as exploratory due to limited sample size

17. Other analyses
→ No subgroup or sensitivity analyses were performed.

Discussion

18. Key results
→ Summarized in relation to the study objective.

19. Limitations
→ Addressed: retrospective design, small sample size, low event rates, confounding by indication, and limited generalisability.

20. Interpretation
→ Findings interpreted cautiously and in the context of existing literature; results considered hypothesis-generating.

21. Generalisability
→ External validity discussed; applicability may be limited to similar healthcare systems with physician-staffed EMS and comparable intoxication patterns.

Other Information

22. Funding
→ No funding reported.

Ethics approval
→ Approved by the institutional review board with waiver of informed consent.

Data availability
→ Data available from the corresponding author upon reasonable request.
