# Supplementary material for: Airway management in acutely poisoned patients with Glasgow Coma Scale less than 9: a retrospective cohort study
Source: BMC Emerg Med. 2026 Aug 6;26:215. doi: 10.1186/s12873-026-01714-5 (PMC13445855; doi:10.1186/s12873-026-01714-5)
Supplement: Supplementary file 2 [file 12873_2026_1714_MOESM2_ESM.docx]

Table S1. Blood gas parameters according to airway-management group

| **Parameter** | **No prehospital intubation** | **Prehospital intubation** | **p-value** |
| --- | --- | --- | --- |
| Sodium, mmol/L | 142.0 (139.0-144.0) | 142.0 (141.0-146.0) | 0.412 |
| Anion gap | 16.1 (14.5-19.4) | 15.6 (14.5-17.1) | 0.883 |
| Lactate, mmol/L | 1.81 (1.24-2.56) | 1.90 (1.70-2.20) | 1.000 |
| Standard bicarbonate, mmol/L | 22.9 (21.2-24.8) | 20.0 (19.9-20.3) | 0.004 |
| Blood glucose, mg/dL | 93 (81.8-100.5) | 93 (82.5-104.5) | 0.945 |

Values are presented as median (IQR). Comparisons were performed using the Mann–Whitney U test.

Table S2. Characteristics of patients requiring delayed intubation within 24 hours

| **Patient** | **Circumstances of discovery** | **GCS at scene** | **Vital signs at scene** | **Prehospital treatment** | **GCS on arrival at emergency department** | **Detected substances** |
| --- | --- | --- | --- | --- | --- | --- |
| 1 | Found in bar | 3 | SpO₂ 85%, BP 167/95 mmHg, HR 65/min, RR 6/min | Antidote therapy with naloxone and flumazenil (no improvement) | 3 | GHB/GBL (clinically suspected) |
| 2 | Suicide attempt, found on cemetery | 8 | SpO₂ 96%, BP 100/62 mmHg, HR 90/min, RR 10/min | No antidote treatment | 3 | Phenobarbital, doxycycline, metoclopramide, metamizole |

Table S3. Vasopressor therapy within 24 hours according to airway-management group

| **Outcome** | **No prehospital intubation (n = 74)** | **Prehospital intubation (n = 10)** | **Effect estimate** | **p value** |
| --- | --- | --- | --- | --- |
| Vasopressor therapy within 24 h, n (%) | 1 (1.4%) | 2 (20.0%) | OR 18.3 (95% CI 1.5–224.3) | 0.036¹ |

¹ Fisher's exact test (two-sided).

Table S4. Distribution of poisoning agents by airway-management group

| **Poisoning agent** | **No prehospital intubation (n = 74)** | **Prehospital intubation (n = 10)** | **Total (n = 84)** |
| --- | --- | --- | --- |
| Ethanol | 27 (36.5%) | 5 (50.0%) | 32 (38.1%) |
| Antidepressants | 1 (1.4%) | 0 (0%) | 1 (1.2%) |
| Illicit drugs | 7 (9.5%) | 0 (0%) | 7 (8.3%) |
| Sedative drugs | 1 (1.4%) | 0 (0%) | 1 (1.2%) |
| Mixed intoxication | 38 (51.4%) | 5 (50.0%) | 43 (51.2%) |
